# How to increase chlamydia testing in primary care: a qualitative exploration with young people and application of a meta-theoretical model

**DOI:** 10.1136/sextrans-2019-054309

**Published:** 2020-05-29

**Authors:** Lorraine K McDonagh, Hannah Harwood, John M Saunders, Jackie A Cassell, Greta Rait

**Affiliations:** 1 Research Department of Primary Care and Population Health, University College London, London, UK; 2 National Institute for Health Research Health Protection Research Unit in Blood Borne and Sexually Transmitted Infections at University College London, London, UK; 3 Institute of Psychiatry, Psychology and Neuroscience, King's College London, London, UK; 4 National Chlamydia Screening Programme, PHE, London, UK; 5 Department of Primary Care and Public Health, Brighton and Sussex Medical School, Brighton, Brighton and Hove, UK

**Keywords:** adolescent, behavioural science, chlamydia infection, general practice, qualitative research

## Abstract

**Objective:**

The objective of this study was to explore young people’s perspectives barriers to chlamydia testing in general practice and potential intervention functions and implementation strategies to overcome identified barriers, using a meta-theoretical framework (the Behaviour Change Wheel (BCW)).

**Methods:**

Twenty-eight semistructured individual interviews were conducted with 16–24 year olds from across the UK. Purposive and convenience sampling methods were used (eg, youth organisations, charities, online platforms and chain-referrals). An inductive thematic analysis was first conducted, followed by thematic categorisation using the BCW.

**Results:**

Participants identified several barriers to testing: conducting self-sampling inaccurately (physical capability); lack of information and awareness (psychological capability); testing not seen as a priority and perceived low risk (reflective motivation); embarrassment, fear and guilt (automatic motivation); the UK primary care context and location of toilets (physical opportunity) and stigma (social opportunity). Potential intervention functions raised by participants included education (eg, increase awareness of chlamydia); persuasion (eg, use of imagery/data to alter beliefs); environmental restructuring (eg, alternative sampling methods) and modelling (eg, credible sources such as celebrities). Potential implementation strategies and policy categories discussed were communication and marketing (eg, social media); service provision (eg, introduction of a young person’s health-check) and guidelines (eg, standard questions for healthcare providers).

**Conclusions:**

The BCW provided a useful framework for conceptually exploring the wide range of barriers to testing identified and possible intervention functions and policy categories to overcome said barriers. While greater education and awareness and expanded opportunities for testing were considered important, this alone will not bring about dramatic increases in testing. A societal and structural shift towards the normalisation of chlamydia testing is needed, alongside approaches which recognise the heterogeneity of this population. To ensure optimal and inclusive healthcare, researchers, clinicians and policy makers alike must consider patient diversity and the wider health issues affecting all young people.

## Background

Chlamydia is the most commonly diagnosed sexually transmitted infection (STI) in England, comprising 49% of STI diagnoses in 2018.^1^ Young people experience the highest rates of chlamydia, accounting for 60% of diagnoses in 2018.^1^ Chlamydia is largely asymptomatic and can result in serious health consequences if left untreated (eg, pelvic inflammatory disease and infertility). Testing and early treatment, therefore, are required to prevent onward transmission and potential negative health outcomes.

### UK context

In the UK, with the aim of controlling transmission through early detection and treatment, the National Chlamydia Screening Programme (NCSP) was established in 2003, recommending that all sexually active individuals under 25 are tested annually or on change of sexual partner.^2^ Although more tests are performed in specialist settings,^1^ the NCSP advocates a wide range of opportunistic testing across settings (eg, primary care settings such as community pharmacies and general practice) and via tests ordered online. While online testing has increased in recent years (11% of total tests in 2017 to 17% in 2018), almost one-fifth (20% in 2017; 18% in 2018) come from primary care (including general practice).^1^

General practice offers considerable testing opportunities: approximately 60% of young men and 75% of young women attend annually^3^ and young people report positive attitudes towards general practice testing.^4^ Furthermore, regular opportunistic testing is facilitated by patients attending for other reasons,^2^ unlike testing via specialist services (including online services) which requires individuals to seek out testing. A recent review identified patient barriers to testing including lack of knowledge, perceived low risk, embarrassment, fear and stigma and facilitators including increased awareness and self-sampling.^5^ It remains unclear how to translate knowledge of these factors into effective interventions and clinical practice. Interventions have had varied results,^6^ the majority demonstrating only modest effects.^7 8^ These results *may* owe in part to a neglect of psychological behavioural theory.

### Behavioural theory

**Figure 1:**
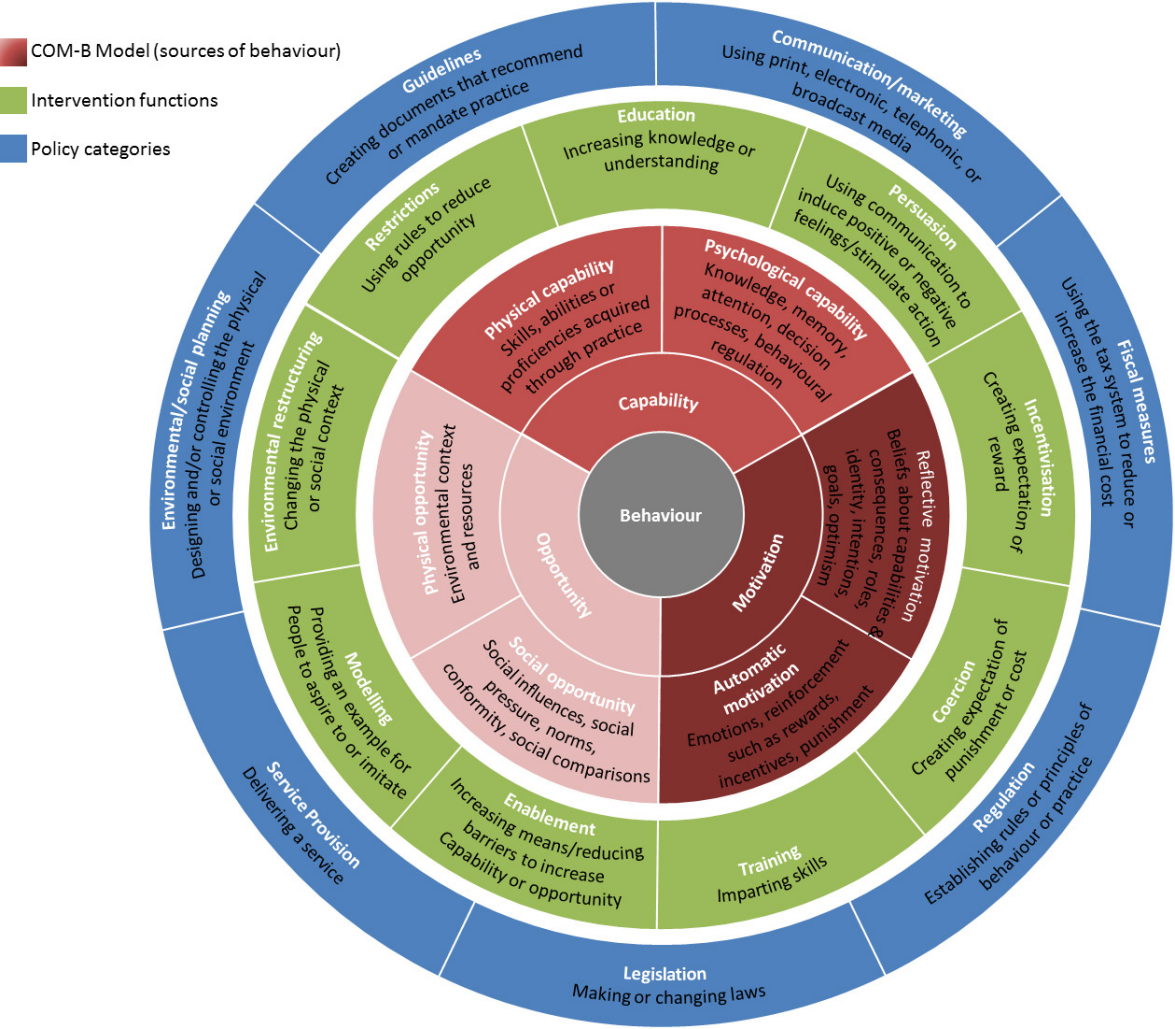
The Behaviour Change Wheel (Michie *et al* 10). COM-B, Capability, Opportunity and Motivation Model of Behaviour.

In order to change a given behaviour and develop effective interventions, it is necessary to have a theoretical understanding of said behaviour.^9^ The Behaviour Change Wheel (BCW) is a meta-theoretical framework and three-tiered tool to construct behaviour change interventions (see figure 1 10 11). The first tier—the Capability, Opportunity and Motivation Model of Behaviour (COM-B)—posits that behaviour results from the interaction of *capability* (psychological (knowledge); physical (skills)), *opportunity* (social (societal influence); physical (environmental resources)) and *motivation* (automatic (emotion); reflective (beliefs about capabilities)). For a person to engage in a specific behaviour (eg, accepting/requesting a chlamydia test), they need to (1) be psychologically and physically able; (2) have the physical and social opportunity and (3) want or need to do the behaviour. The second tier (Intervention Functions) outlines nine categories through which behaviour can be changed: education, persuasion, incentivisation, coercion, training, restriction, environmental restructuring, modelling and enablement. The third tier (Policy Categories) details seven policy strategies to support interventions functions to bring about behaviour change: communication/marketing, guidelines, fiscal measures, regulation, legislation, environmental/social planning and service provision.

The advantage of the BCW is this framework helps to identify a range of factors influencing behaviour (tier one), and potential intervention (tier two) and implementation (tier three) options that could help support change in the given behaviour. The BCW has already supplied the foundation for several health-behaviour interventions;^12–18^ thus, applying the BCW to general practice chlamydia testing could aid intervention development to increase testing.

### Current study

The purpose of this study was to explore: (1) barriers to chlamydia testing in general practice; (2) potential intervention functions to overcome identified barriers and (3) potential policy categories to support intervention functions from the perspective of young people.

## Method

### Participants

Semistructured individual interviews were conducted with 16–24 year olds in the UK. Purposive and convenience sampling methods were used: snowballing, social media advertisements (eg, *Facebook*, *Twitter*, *Instagram*) and promotions by organisations and charities. Over 600 groups from across the UK were contacted (eg, youth choirs, student unions, community colleges, football clubs, youth political parties, sexual health charities). The aim was to reach a diverse population with representation across demographics (sex, age, ethnicity, education level and chlamydia testing experience).

### Procedure

Interviews were conducted in person (n=9) or via telephone (n=19). Participants could choose to participate in person or via telephone to allow people from a variety of locations to take part and to enhance participant comfort. Some individuals feel more comfortable discussing sensitive topics while being anonymous. Phone interviews can increase participants’ perceptions of anonymity, which may therefore enhance the quality of the data produced.^19–25^ Notably, a direct comparison of transcripts from in-person and phone interviews found that both produced similar data.^24^

For efficiency, the participant information sheet, consent form and demographic questions were emailed to participants 1 week in advance of the interview. An interview schedule guided discussions (online supplementary file 1) which was iteratively developed through literature reviews, expert consultation and patient and public involvement. A £10 shopping voucher was offered to all as an incentive. Data collection continued until data saturation was reached.^26^

10.1136/sextrans-2019-054309.supp1 Supplementary data

### Data analysis

On average, interviews lasted 31 min (range=19–42 min). Interviews were digitally recorded and transcribed verbatim. Identifying information was removed.

Analysis and data collection took place simultaneously to enable emerging topics to be investigated in future interviews. Data were subject to inductive thematic analysis^27^ and mapped onto the BCW. The following procedure was employed: (1) The first transcript was read several times to increase data familiarity. Notes were made regarding insightful comments. (2) Transcripts were coded using NVivo12. (3) Once all transcripts were coded, a list of codes was constructed and sorted into provisional themes/subthemes. (4) The thematic list was reviewed and refined; codes and transcripts were revisited to explore whether themes/subthemes satisfactorily represented the data. (5) A final list of themes/subthemes was created and mapped onto the BCW (see online supplementary file 2 for a thematic overview).

10.1136/sextrans-2019-054309.supp2 Supplementary data

To ensure rigorous analysis and to validate findings,^28^ all transcripts were analysed independently by two authors (LM/HH), and other coauthors (GR/JS/JC) analysed a subset of transcripts (ie, two transcripts). Resultant codes and themes were discussed and compared at regular data-analysis meetings to ensure data were represented and displayed in a meaningful manner. No major differences were found; minor discrepancies were discussed. Consequently, BCW categorisation was conducted by the first author (LM) following expert guidelines^10 11^ in consultation with team members at data-analysis meetings (JMS/JAC/GR). Discrepancies were resolved by consensus.

## Results

Twenty-eight interviews were conducted. Participants were predominantly heterosexual (n=19), White British (n=22), female (n=18), students (n=11) and had previously had a chlamydia test (n=17) (online supplementary file 3). Two participants were previously diagnosed with chlamydia. A thematic overview of all findings is provided in online supplementary file 3. The identified barriers to testing (see table 1), interventions functions (see table 2), and policy categories (see table 3) are described in turn.

10.1136/sextrans-2019-054309.supp3 Supplementary data

**Table 1:** Behavioural diagnosis of chlamydia testing with the COM-B Model: barriers to testing in general practice with illustrative quotations

COM-B components	Themes and subthemes		Illustrative quotations
*Physical capability* Skills, abilities or proficiencies	Self-sampling—collecting and sealing the sample (in)correctly		Q1.1: ‘Even doing this swab… I don’t know how wrong you can do it, but sometimes you still worry like!’ (P01, female, 24 years) Q1.2: ‘People might worry they’ve not done the test right, especially if they’re younger and not done them before. I actually did do one wrong once! I told the nurse about it and she said, ‘What did you do? Did you stick it up your arse?’ But it is possible to do it wrong, I think medical professionals maybe don’t really realise.' (P11, female, 21 years) Q1.3: ‘If I was doing it, I would be quite nervous about messing it up and getting it wrong. I’d prefer to be able to talk to the nurse or doctor and ask them questions about it first, and even after you do the test, you could still have questions, you know. ' (P09, female, 17 years)
*Psychological capability* Knowledge, memory, attention, decision processes, behavioural regulation	Lack of information and awareness	Asymptomatic	Q1.4: ‘If they don’t have symptoms and they don’t think it’s anything bad, but they don’t know that it can lead to bad thing.' (P07, female, 20 years)
		Risk of transmission	Q1.5: ‘I don’t think a lot of people know enough about chlamydia, like the dangers and stuff… not knowing how they could get it, I guess, if they don’t really know much about it.' (P27, female, 16 years)
		Testing process	Q1.6: ‘Before I ever got one done, you think it is a bit kind of intrusive because you don’t know what it is.' (P03, female, 22 years) Q1.7: ‘Isn’t it just like where they prick your finger and test the blood?' (P18, male, 19 years)
		Ease of treatment	Q1.8: ‘But I know when I was younger when I used to think of sexually transmitted diseases and infections, I used to think that’s it, that’s the end of it; if you get one, you’re stuck with it for life.' (P03, female, 22 years)
		Availability in general practice	Q1.9: ‘I didn’t even know myself you could do that at the GP…' (P28, female, 16 years) Q1.10: ‘Maybe I’m naïve, but I didn’t even know that was something you would get at the GP really. I suppose I don’t know where you would go for it, obviously there’s clinics, but I suppose it’s not something you, well I would relate to the GP, going for a sexual health test.' (P12, male, 23 years)
*Reflective motivation* Beliefs about capabilities and consequences, roles, identity, intentions, goals, optimism	Testing not a priority		Q1.11: ‘I probably did do something I shouldn’t have done, and then realising that I should probably go, but not quite having the time. And then you might forget about it for a few weeks.' (P01, female, 24 years)
	Perceived low risk	Sexual invincibility	Q1.12: ‘It’s one of these things where people think that it’ll never happen to them, where, you know, obviously it can happen to anyone. I think people are a bit oblivious about it.' (P08, female, 23 years)
		Relationship status (monogamy)	Q1.13: ‘Because I had a boyfriend I was like it’s very unlikely that I have chlamydia, or so I thought…' (P11, female, 21 years) Q1.14: ‘I’m not very much at risk of having chlamydia because I have a long term girlfriend, I’m faithful and all that stuff, so… well, I was going to say I haven’t had any reported symptoms in the past, but as it’s symptomless, it would be difficult to find out… But I think my girlfriend got tested and she’s clean, so I just assumed that, you know, so am I.' (P16, male, 23 years)
		Belief chlamydia is not serious	Q1.15: ‘That’s (chlamydia) not the one I’m worried about, like I’ve had it once, and I suppose I’d be more worried about the other STIs.' (P11, female, 21 years) Q1.16: ‘For me it would seem a little bit pointless to go and get it done just for chlamydia, because I don’t worry about chlamydia specifically.' (P17, male, 21 years)
*Automatic motivation* Emotions, reinforcement such as rewards, incentives, punishment	Embarrassment	Knowing their GP	Q1.17: ‘People are quite embarrassed about going to their GP because it’s somebody they know quite well… you know your GP, so to have that embarrassment of going through that stuff with them, it can be a bit uncomfortable sometimes.' (P15, female, 24 years)
		Being seen	Q1.18: ‘It might be like an uncomfortable situation, just in case you see someone you know or like, along those lines.' (P25, female, 18 years)
		Procedure as invasive	Q1.19: ‘But I think maybe if it was a swab test - if it’s just chlamydia, like I might be put off because that’s a bit intrusive as a test.' (P06, female, 24 years)
		Having to take clothes off	Q1.20: ‘Well, I mean I’m not entirely sure how you get tested for some stuff, you know. No one really wants to walk in to the doctor’s and have to drop your pants in front of them, it wouldn’t be the most memorable day of your life. So I think embarrassment definitely is probably the number one problem.' (P26, male, 20 years)
	Fear	Long term consequence	Q1.21: ‘Because there’s fear about this silent killer that is chlamydia, which shows no symptoms until you try to make babies and they don’t happen' (P16, male, 23 years)
		Expectations of stigma	Q1.22: ‘Some people probably would be quite afraid, maybe some people see the stigma around it, so would feel quite scared going for that reason.' (P17, male, 21 years)
		Unknown	Q1.23: ‘I was scared because I didn’t know. I was scared because I was like, oh, I don’t know what I have to do.' (P03, female, 22 years)
		Positive result	Q1.24: ‘Yeah, because I suppose giving out figures of how dangerous STIs can be and stuff, because what I was going to say earlier was, I guess, like one of the reasons some people don’t get tested is because they’re maybe scared of the results.' (P12, male, 23 years)
	Guilt		Q1.25: ‘The hesitating element is that you know you’ve done something wrong, either you’ve cheated on your girlfriend and you think you might have it, or maybe you were with a girl that you maybe had a one night stand.' (P16, male, 23 years)
*Physical opportunity* Environmental context and resources	UK primary care context	Strained system	Q1.26: ‘It’s just a problem with the whole way that the NHS is being treated at the moment, with just cutting and cutting and cutting, which means that actually GPs don’t have the time and they don’t have the energy, like, extra resources to do extra, like, promote these kind of things, when they need to be.' (P02, female, 24 years)
		Registration	Q1.27: ‘I think the problem with the GP would be that I have to be registered with one and I’m not.' (P16, male, 23 years)
		Getting appointments	Q1.28: ‘It’s really hard to get an appointment at my GP.' (P11, female, 21 years) Q1.29: ‘Appointments are really hard to get at GPs anyway… So to go at all requires getting up one morning and ringing at eight, and also managing to get time off work… So actually to go to the GP at all is an ordeal.' (P01, female, 24 years)
		Lacks urgency	Q1.30: ‘At my GP, you either get an appointment about a month in advance or, if you call up on the day, then you can be on the phone for like an hour waiting for it to clear. Then you feel like if I’m going to wait that long, it needs to be for an emergency not just for a chlamydia test. Or you can go down at 8.45 am and literally queue up outside, which again, for a chlamydia test feels like a bit much. Because it’s not like necessarily, oh, my hand won’t stop bleeding(!) (slight laugh) and something immediately is going to happen. I guess with chlamydia, it feels like something you can put off.' (P02, female, 24 years) Q1.31: ‘It just might be a general GP problem, when you go to a GP especially when it’s not urgent, you can have a 2 week wait to get an appointment… so sure it doesn’t have symptoms so you mightn’t bother and forget it.' (P25, female, 18 years)
		Time constraints	Q1.32: ‘I suppose if you’re sick and you go to the GP, the main focus is getting rid of your cold or your chest infection, so there’s not time to think about chlamydia.' (P11, female, 21 years) Q1.33: ‘And also sometimes I’ve been to the GP where it seems like they’re pressed for time and they’re trying to rush through everything. So if they asked me for a chlamydia test and it became clear that they kind of were pushed for time, then I’d be like, ‘Oh, no, it’s OK’.' (P11, female, 21 years)
	Location of toilet (links to embarrassment)		Q1.34: ‘For me, I guess, like the only thing that ever really makes me feel slightly uncomfortable is when the toilets are in the patient waiting room… I’ve experienced that once or twice, and when you have to go and do something yourself, like a swab or pee somewhere, and they just give it to you, it’s like you have to walk in there in front of everyone and walk out.' (P04, female, 24 years)
*Social opportunity* Social influence, pressure, norms, conformity, comparisons	Stigma	Sex taboo	Q1.35: ‘It’s funny because it is your doctor and they are supposed to do your health issues like that, but I think with sex and sexual health, people often need, eh it’s like you feel it’s that taboo thing.' (P02, female, 24 years)
		Presumed promiscuity	Q1.36: ‘Think some people associate a stigma with going to get a test.' (P05, male, 23 years) Q1.37: ‘I think it’s just the whole stigmatisation around chlamydia and having chlamydia. I feel like if someone had it, they’d rather not know that they had it, because it’s so stigmatised.' (P20, female, 19 years) Q1.38: ‘Just the stigmatisation around having chlamydia, because if you have it, people assume that you sort of like sleep around loads and stuff, but it’s not always because of that… but that’s what’s stopped me in the past.' (P20, female, 19 years)
		Judgement from HCP	Q1.39: ‘I think a lot of young people think that health practitioners, they feel like they’re getting judged by them because of their lifestyle and the way they kind of go about things.' (P15, female, 24 years).
		Judgement from receptionists	Q1.40: ‘It can be quite difficult if they say, ‘What’s the reason for your appointment?’ and trying to explain it to a receptionist, they maybe think oh, she’s a receptionist, I can imagine some people thinking, that receptionist isn’t going to like it, she’s going to know what I’m saying, and she’ll think I’m really gross.' (P13, female, 22 years)
		Younger age	Q1.41: ‘I guess because if it’s ranging from ages 16 to 24, I guess that depends on your age, because if you’ve got a 24 year old, I think there’s less stigma attached; it’s only for a 16 year old I think, yeah, I think there is still a social stigma… people worry about getting tested.' (P14, male, 24 years) Q1.42: ‘It’s (chlamydia) got a lot of stigma, I’d definitely say it’s 100% stigma, definitely.' (P23, male, 17 years)
		Never tested	Q1.43: ‘Like, when you’ve not tested before, you just feel even more stigma and embarrassment about it, then once you’ve done it once, I think it eases then, a bit.' (P25, female, 18 years)
		Sexual orientation	Q1.44: ‘The issue is if they’re not out to family, or friends, they might not be comfortable coming out to some member of staff.' (P25, female, 18 years) Q1.45: ‘I would be slightly more worried about the doctor or the nurse practitioners turning their noses up slightly.' (P09, female, 17 years) Q1.46: The last time I was in for a non-sexual related reason, and they asked me something sexually related, about my sexuality, and I felt hostility in the question. And that was quite insulting really. So I think, yes, people being hostile to sort of LGBT definitely is slightly off-putting… So I went in for a non-sexual related issue and she thought it could have been caused by gay sex. And had a rant about the damage it gave you, that people can do to themselves, through gay sex and stuff. And it was quite an aggressive thing. There was no support and no nice comment. It was just how badly we could hurt ourselves and stuff like that.' (P26, male, 20 years) Q1.47: ‘People maybe don’t want to share that they’re LGBT with their GP or whatever. I suppose that’s why I like (GUM clinic), a lot of people go there instead of going to the GP… they probably feel more comfortable there. Everyone that’s going there is LGBT and the people that are testing them are maybe more familiar with LGBT specific sexual health issues.' (P12, male, 23 years)

COM-B Model, Capability, Opportunity and Motivation Model of Behaviour; GP(s), general practitioner(s); LGBT, lesbian, gay, bisexual, transgender; NHS, National Health Service; P, participant number; Q, Quote number; STI, sexually transmitted infection.

**Table 2:** Potential intervention strategies: Interventions options and illustrative quotations to overcome barriers to chlamydia testing in general practice

Intervention options	Themes and subthemes		Illustrative quotations
*Education* Increasing knowledge or understanding	Increase information and awareness	Transmission	Q2.1: ‘I think it’s a case of both making people aware of chlamydia itself and what the risks are and what the dangers are.’ (P25, female, 18 years)
		GP testing	Q2.2: ‘Just reminding me that I can get it done there I guess, because I forget that I can get it done there as well.’ (P07, female, 20 years) Q2.3: ‘For me personally I would just think if we know that the GP actually does it, then we can actually go there.’ (P19, female, 19 years)
		Testing process	Q2.4: ‘I think they just need to make it out to people that it’s not a real intrusive test, you know, you can do it yourself; like it takes, what, two seconds to do. I think that should probably be highlighted more because, like I said, no-one really said that to me before I was getting it done.’ (P03, female, 22 years)
		Consequences of not testing	Q2.5: ‘Mentioning that chlamydia can lead to infertility because I think a lot of people don’t know that.’ (P07, female, 20 years) Q2.6: ‘It’s only going to benefit you if you get it tested, I think it’s a good question (offer testing) for a doctor to ask, just to make sure, purely because I don’t think enough people actually get tested, they don’t see the benefit of just doing it.’ (P21, female, 22 years)
		Ease of treatment	Q2.7: ‘Because I think a lot of people think it’s going to be invasive and it’s going to be with them for life, but it’s not, it is easily treated.’ (P13, female, 22 years). Q2.8: ‘Having the right information about, about how the test is taking place, and also, yes, so reassurance and it’s not such a big deal to get treated and things like that.’ (P26, male, 20 years)
	Target younger ages groups		Q2.9: ‘Increasing the awareness of it - especially the sort of younger ages - maybe when you get a bit older, you’re more aware of what’s going on, but maybe in your teenage years you’re less sort of savvy about how to go about getting a test.’ (P05, male, 23 years) Q2.10: ‘Stigma, if you were taught from a younger age about these things, you’d probably start feeling more open about them… I think we were told very much what they are but not what all the, erm … you know, what the sort of emotional side of it is, we were not taught that at all.’ (P01, female, 24 years) Q2.11: ‘You also just kind of, you’re young, so you fear the worst, because you just don’t know.’ (P28, female, 16 years) Q2.12: ‘Also again sort of the whole stigma surrounding it, when I was younger that put me off (testing).’ (P18, male, 19 years)
	School-based education	Inclusion early in education	Q2.13: ‘If it was taught earlier in school that you should be doing this yearly, or you should be doing this after every sexual partner.’ (P14, male, 24 years) Q2.14: ‘But also education at school is going to be a massive thing, because I didn’t have that sort of education and I’ve been trying to sort of get help that in the community, and that makes a difference because you’re sort of aware of how lacking in education kids are.’ (P18, male, 19 years) Q2.15: ‘I think we were told what they (STIs) are but not what all the, erm … you know, what the sort of emotional side of it is, we were not taught that at all.’ (P01, female, 24 years)
		Relevance for all	Q2.16: ‘My sex education was shocking… it wasn’t really informative. It didn’t really talk about things you could pick up during sex. It was, it was mainly, ‘Oh this is a period,’ and actually crack on, okay, not very helpful.’ (P26, male, 20 years)
*Persuasion* Using communication to induce positive or negative feelings, or stimulate action	Framing of communication	Testing as responsible behaviour	Q2.17: ‘You should almost be proud of going to get checked because you’re doing the responsible thing. Even if you’ve done a silly thing, you know slept with someone and had unprotected sex with someone.’ (P12, male, 23 years)
		Testing as healthy behaviour	Q2.18: ‘I think reassurance that it’s not something to be ashamed for, that people go through it, no matter the age and it’s obviously healthier for you, in the long run, to do it.’ (P26, male, 20 years)
		Moral obligation to others	Q2.19: ‘I think it’s also about not having it yourself, so you know you’re not the person that’s passing it on; it’s not just about catching it.’ (P01, female, 24 years)
		Positive reinforcement	Q2.20: ‘When you go and get a test, someone should say that’s a good thing you’re getting a test and feel free to come in again… you’d get some positive reinforcement to sort of tell you that it’s a good thing that you’re doing.’ (P05, male, 23 years)
		Conformity - everyone does it	Q2.21: ‘Reiterate the fact that everybody gets tested at some point, and that it’s not a personal dig at you! You know it is just that everybody needs to get tested at some point… it is just that everybody of all ages and all backgrounds gets tested at some point.’ (P15, female, 24 years)
	Use imagery or data to alter beliefs		Q2.22: ‘You’ve got to give them the fear a little bit… they don’t want to be, but maybe you have to scare them a little bit. there’s always the option to either have horrible pictures or, you know, statistics based on that sort of thing and just make sure that they’re really well publicised.’ (P17, male, 21 years)
	Challenge perceptions of chlamydia		Q2.23: ‘If you do have chlamydia, it doesn’t mean you’re going to be sick for the rest of your life, which I think a lot of younger people might think that! It’s not like that, especially nowadays, but, it definitely should be highlighted more that if you don’t get treated, it will actually affect you.’ (P03, female, 22 years) Q2.24: ‘I think just trying to relinquish the taboo over it a little bit, I guess. People, I think there’s just so much emphasis on like don’t get STIs; it’s like don’t get them, they’re horrible! And it’s actually like, well, chances are you might get it and that’s OK, because it’s really treatable. If you can try and protect yourself from it, that’s the first option, but if you do get it, we can treat it, as long as you test it and you know that there is that option of easily treating it. Prevention is always better than cure, but I just think, not normalising it but in a sense, maybe normalising it and just going, like: This happens to people, don’t feel ashamed, come and get treated, because we all have sex!’ (P04, female, 24 years) Q2.25: ‘I feel like I understand that it’s quite dangerous and I do get anxious about it if I don’t (get tested), so if people don’t even realise that it’s a dangerous thing and they’ve put themselves at risk, if they think it’s not important to get tested, then that definitely needs to be changed, yeah.’ (P22, male, 18 years)
*Environmental restructuring* Changing the physical or social context	Flexible appointments		Q2.26: ‘I think it would be easier if they didn’t have to book an appointment in advance, if they could just drop in whenever they needed to. Or maybe if the hours weren’t restrictive like they are at some of the clinics… So if it was kind of available most of the time, I think that would help as well.' (P07, female, 20 years) Q2.27: ‘Being more flexible with timings and stuff, and being able to rock up and get an appointment that day, or wait around and get an appointment like at a walk-in; just it being a bit less structured and ease of being able to get in and out essentially…I mean, walk-in sessions are pretty paramount.' (P18, male, 19 years)
	Toilet location		Q2.28: ‘I’ve experienced that once or twice, and when you have to go and do something yourself, like a swab or pee somewhere, and they just give it to you, it’s like you have to walk in there in front of everyone and walk out. And most people are really good, because they have toilets next to the rooms, like the practice rooms and stuff.' (P20, female, 19 years) Q2.29: ‘I had to give a sample and the toilet was right in the patients’ waiting area… it’s that sort of lack of privacy that needs to be addressed.' (P28, female, 16 years)
*Modelling* Example to aspire to/imitate	Example provision		Q2.30: ‘Give a link to an instructional video or something like that, that kind of just shows it (testing) as well… And with something like a swab you could use a model, like a plastic kind of anatomical model to just show how to do it and things like that, so that it’s kind of physically demonstrated.' (P24, female, 22 years)
	Credible sources		Q2.31: ‘Like how they did with Prince Harry and the HIV testing, if they did something similar but for chlamydia…hm but I suppose who’d want to be the face of chlamydia?' (P10, male, 24 years) Q2.32: ‘If it was on TV shows or something they might think about it a bit more, and if you saw your idols doing it (testing), you might do it too.’ (P07, female, 20 years)
	Friend referrals		Q2.33: ‘Yeah, posters in the place, give out a leaflet and then they can go and give it to their friends or whatever. Encourage them to get tested and pass it (the leaflet) on.’ (P12, male, 23 years) Q2.34: ‘If you could get people to go to testing with their friends, sorta make a day out if it, I think that could help, that’s what helped me before.’ (P15, female, 24 years)

GP, general practitioner; P, participant number; Q, Quote number; STI, sexually transmitted infection.

**Table 3:** Potential implementation strategies: policy categories and illustrative quotations to support intervention functions and overcome barriers to chlamydia testing in general practice

Policy categories	Themes and subthemes		Illustrative quotations
*Communication and marketing* Using print, electronic, telephonic, or broadcast media	Circulating information on chlamydia	Community adverts	Q3.1: ‘Increasing advertising, like at universities and things, increasing awareness of how you can get tested; just go along to your GP and get one.’ (P05, male, 23 years)
		TV and radio advertisements	Q3.2: ‘I think TV adverts would actually get people to sign up, because then I think… it would suddenly cross their minds that they might have it… Because I think the main problem is that people don’t think, they just don’t think about it.’ (P18, male, 19 years)
		General practice posters and leaflets	Q3.3: ‘Put up posters in the place [general practice], give out a leaflet and then they can go and give it to their friends or whatever… Encourage them to get tested; pass it on.’ (P12, male, 23 years)
		Social media	Q3.4: ‘Well, looking at the sort of age range, you’ve obviously got to look at what they’re accessing, so social media.’ (P13, female, 22 years)
	Reminder letter		Q3.5: ‘Like how they send out letters for smear tests when you’re 25 - if they did something similar when you’re 16 or 17 or whatever, saying that you can at any time go and get a chlamydia test… or a yearly letter to people or a yearly phone call, just reminding people that they can go and get it done, I think that would help a lot.’ (P07, female, 20 years)
	Clear instructions for self-sampling kits		Q3.6: ‘I think just make sure the instructions are really clear and provide reassurances, like, it’s really hard to do this wrong, don’t worry, with pictures and stuff… yeah, it’s very important to have clear instructions.’ (P11, female, 21 years)
*Service provision* Delivering a service	GP offering testing		Q3.7:‘If you were going to see your doctor about something and it was offered to you, I think far more people would be willing to take it. If it was something that someone said that you can also do this and have a test, then people would probably do it. But if it’s somebody going out of their own way to go and get themselves tested, then I think it’s actually just some people are, maybe even me, just lazy!’ (P14, male, 24 years)
	Option of alternative staff	Staff unknown to patient	Q3.8: ‘You know your GP, so to have that embarrassment of going through that stuff with them, it can be a bit uncomfortable sometimes… Whereas if it’s just a nurse who you don’t really know that well or haven’t really met before, it’s kind of easier to deal with.’ (P15, female, 24 years)
		Gender of staff (relatability)	Q3.9: ‘This is a bit picky, but I will only go and see a female GP for anything I’ve got wrong with me… So maybe get someone like the same sex… or give them the option… because you sort of like think they understand more of what you’re going through, because obviously, you go to a male GP about summat [sic] and you just feel they don’t understand, so you can’t tell them everything.’ (P27, female, 16 years)
	Alternative sampling methods	Preference for urine sample	Q3.10: ‘I think maybe if it was a swab test, if it’s just chlamydia, like I might be put off because that’s a bit intrusive as a test, but if you could just do the wee sample then I don’t think it would put me off, no.’ (P06, female, 24 years)
		Preference for self swab	Q3.11: ‘I think if it’s a swab one, then it’s definitely better to do it yourself. It’s sort of like quite a private thing that you can do yourself, yeah, privacy is important’ (P01, female, 24 years)
		Preference for HCP swab	Q3.12: ‘I always just want the nurse to do it, to do all the swabs and things just to make sure that it was done right’. (P08, female, 23 years)
	Discreet systems in practice	Drop-box	Q3.13: ‘If they had like the tests in the toilets or something, people might just do it whilst they’re there. If there’s like a drop-box and then at least you don’t have to talk to somebody.’ (P06, female, 24 years)
		Collection point	Q3.14: ‘Having them available for you to just pick up and take one, and do one whenever you want.’ (P22, male, 18 years)
		Postal kits	Q3.15: ‘I actually thought the kits that you can do in the post are really good, if they still offer them, because then you don’t have to go anywhere. I think you could just put them in the letterbox as well, actually. Yes, you’d only have to go to the post office. (slight laugh) Yeah, I think that would be good for people who are too busy.’ (P07, female, 20 years)
	Home self-sampling		Q3.16: ‘Even those kind of home kits that you can just drop it into your doctor, I think that’s kind of good because people prefer to do it in their own home.’ (P03, female, 22 years) Q3.17: ‘I think it’s a pretty good option to have. I think you’ve got to give people the different options though, cos I personally wouldn’t like to do it that way, I wana get it done there and then. But we’re all different aren’t we.’ (P05, male, 23 years)
				Online testing via GP website	Alternative option	Q3.18: ‘I think the online thing is really good. I think if I was leaving, if I went to the doctor and if was on my way out and they said, ‘You can do this test online,’ that would be a real, like, oh, wow, that’s so good! I just think that’s a really, really good idea.’ (P23, male, 17 years)
	Maintain in-person options	Q3.19: ‘I think there’s something about going and speaking to a person, even if you do the test yourself, like if you have any questions about it, even just stupid questions like how likely am I to have this or when will I get the results back? I just like speaking to someone.’ (P11, female, 21 years)		
	Young person’s health check		Q3.20: ‘I think if it was generally like a sexual health thing, like when teenagers become sexually active, from like maybe the ages of 16 onwards, it just became a routine thing. You do a health check, check your lung functions, your physical health and then on top of that, as part of the natural process, you ask about kind of sexual health. You know, like an elderly person would get their prostate examined; a younger person could get a sexual health exam as part of it.’ (P16, male, 23 years)
	*Guidelines* Creating documents that recommend to mandate practice.	Offered in all consultations	Offer during every visit	Q3.21: ‘The GP could just ask you every time you went, like, ‘Do you want one?’ you know, so they have a lot of questions, but your GP, you know, if they just said it every time, I’m sure they’d get quite a lot of people to take them regularly, you know, having your GP offering it to you as part of your check-up, that could be quite effective.’ (P17, male, 21 years)
Approach appropriately			Q3.22: ‘I guess it would depend how they offered it. It might make me feel a bit worried, like they were implying that I had chlamydia.’ (P28, female, 16 years)
Non-judgmental communication			Q3.23: ‘You want someone with zero judgement who will just come and treat it like a normal thing. Otherwise people would be put off from going again if you get someone who makes a comment that you don’t really like.’ (P05, male, 23 years)
Standard question			Q3.24: ‘When you’re with the medical professional, and they propose it as a very commonplace and comfortable thing, and common and easy thing to do… If that [chlamydia test] became just a standard question to ask for a demographic that was particularly at risk, then at least it’s kind of like when you go to the supermarket and they ask if you want a bag, and you spend maybe three seconds thinking yes or no. So normalising it a bit more is very important.’ (P16, male, 23 years)
Do not single people out			Q3.25: ‘I’d feel a bit weird if it felt like they were singling me out, but if they just kind of offered it as, hey, we’re just offering this for everyone type thing, I would feel absolutely fine about it.’ (P24, female, 22 years)
Personable, friendly staff			Q3.26: ‘I think personally I would prefer someone quite outgoing, talkative, you know, calms you as soon as you step into the room, tell a joke maybe. I think that’s personally the way I would prefer it, rather than going in there and the person be massively serious and barely talk to you. It would definitely be better, and I’d be more likely to go back again if it was like that to be honest.’ (P26, male, 20 years)

GP(s), general practitioner(s); P, participant number; Q, Quote number.

### Barriers to testing

The identified themes and sub-themes for barriers to testing, and illustrative quotes, are provided in table 1.

#### Physical capability (physical skills)

Several female participants were concerned about performing self-collected vulvovaginal swabs, particularly doing something wrong and impacting result accuracy (see table 1, quote number 1.1: Q1.1). Many viewed self-sampling positively, however, and the opportunity to ask questions and raise concerns with a healthcare professional (HCP) was important for younger, less experienced and first-time testers (Q1.2; Q1.3).

#### Psychological capability (knowledge)

Over half of the participants discussed their—and others’—lack of chlamydia knowledge and awareness, for example, that chlamydia could be asymptomatic (Q1.4), ways of transmission (Q1.5), what testing involves (Q1.6, Q1.7) and ease of treatment (Q1.8). Some were not aware that testing is available in general practice (Q1.9; Q1.10).

#### Reflective motivation (evaluations, beliefs and plans)

Testing was not a high priority for many; participants cited being ‘*too busy’* or forgetting to test (psychological capability; Q1.11). This stemmed from perceptions of low, or no, personal risk of acquiring chlamydia. Many discussed ‘*sexual invincibility’* and viewed themselves as impervious to chlamydia, regardless of sexual behaviour (Q1.12).

Beliefs that monogamous relationships meant no risk of chlamydia were salient. However, one participant shared how she acquired chlamydia during what she believed to be a mutually monogamous relationship (Q1.13). Others had not considered that asymptomatic infections could pre-date relationships if both partners had not tested (Q1.14).

Some felt chlamydia was not serious and perceived other STIs as more severe; this was especially apparent for participants who previously had chlamydia (Q1.15). A few participants wanted to be tested for all STIs, not ‘*just’* chlamydia; they felt that if there is the potential for chlamydia, there also exists potential for infection by other, ‘*more serious’*, STIs (Q1.16).

#### Automatic motivation (emotions and impulses)

Emotional responses (embarrassment, fear and guilt) were problematic for many. Young people could feel uncomfortable discussing sexual health in person, especially if they have known their general practitioner (GP) for a long time (Q1.17). Embarrassment could result from being seen by someone known to them (friends, neighbours; Q1.18 or from the testing procedure itself (perception that testing is invasive (Q1.19) and required showing genitals to HCPs (Q1.20)). Fear resulted from the long-term effects of chlamydia on infertility (Q1.21), expectations of stigma (social opportunity; Q1.22), not knowing what the testing process involves (psychological capability; Q1.23) and the possibility of receiving a positive test result (Q1.24). One participant discussed guilt as a barrier due to infidelity during a monogamous partnership and using denial (and therefore not testing) as a coping mechanism (Q1.25).

#### Physical opportunity (afforded by the environment)

Many felt the UK National Health Service (NHS) primary care context reduced physical opportunities for testing through structural barriers (Q1.26). One participant was not registered with a GP (Q1.27), making appointments can be difficult (Q1.28) and participants were unwilling to take time off work/study to attend (Q1.29). Although some practices offered walk-in clinics, these were usually for urgent cases. Several felt that there was no urgency with chlamydia (especially if asymptomatic) so testing can be delayed (Q1.30, Q1.31). Time within appointments is limited; people usually present with multiple concerns and their original concern gets priority (Q1.32). Several believed GPs were ‘*overstretched’*, felt rushed during appointments and did not have the space to discuss sexual health (Q1.33). The toilet location was a deterrent if located in an area near other patients (embarrassment; Q1.34).

#### Social opportunity (afforded by the cultural milieu)

Social stigma stemmed from the taboo nature of sex and, hence, sexual health (Q1.35). Stigma was associated with the need to test (Q1.36) and having a positive diagnosis (Q1.37), due to assumptions of promiscuity (Q1.38). Although several acknowledged that testing and diagnoses do not equate to promiscuity, they felt strongly that other people held these beliefs and feared judgement from staff for their sexual behaviour (Q1.39, Q1.40). Younger participants and those who had never tested were particularly affected, but the impact of stigma lessened with age for some (Q1.41, Q1.42, Q1.43).

Several gay, bisexual and lesbian participants discussed how sexual orientation stigma affected willingness to be tested in general practice. If they were not yet out to family/friends, they were uncomfortable disclosing their sexual orientation to HCPs (Q1.44). Of those who were out, few were comfortable sharing this with HCPs due to expected (Q1.45) and actual (Q1.46), experiences of judgement. Consequently, several preferred sexual health clinics which they felt were better equipped for their specific concerns (Q1.47).

### Intervention functions

The identified themes and sub-themes for intervention functions, and illustrative quotes, are provided in table 2.

#### Education (increasing knowledge)

The majority expressed the need for more public awareness and information on the methods of transmission (Q2.1); existence of free general practice testing (Q2.2, Q2.3); testing processes (Q2.4); testing benefits and consequences of not testing (eg, infertility; Q2.5, Q2.6) and ease of treatment (Q2.7, Q2.8). Participants of all ages felt that younger age groups would benefit the most from education (Q2.9, Q2.10, Q2.11, Q2.12). This could be supported by school-based education (Q2.13), which most felt was either absent from their curriculum (Q2.14) and, when it was included, several felt it lacked specific elements that they perceived to be valuable (eg, emotions, pleasurable sex, STIs; Q2.15). Of the sexual minority participants who had received sex education, all felt the information they received was inappropriate for them—typically, the focus was on avoiding pregnancy (Q2.16).

#### Persuasion (communication to induce feelings or stimulate action)

Participants noted that communication could induce positive and negative feelings to encourage testing. Positive communication should frame testing as a responsible (Q2.17), healthy behaviour (Q2.18) and a moral obligation to others (Q2.19) to prevent transmission. Communication does not need to be explicit; subtle messages could be effective; for example, positive verbal reinforcement (praise; Q2.20) by HCPs when discussing testing could use people’s desire to conform by focusing on the message that ‘*everyone does it’* (improving normalisation; Q2.21). Imagery (eg, depictions of chlamydia consequences) and data (eg, statistics on those infected) could be used to induce negative feelings (fear), to stimulate action (Q2.22). Communication should challenge beliefs and perceptions of chlamydia testing treatment, the long-term impact of undiagnosed infections which may induce fear and, potentially encourage testing (Q2.23, Q2.24, Q2.25).

#### Environmental restructuring (changing physical or social context)

Participants wanted more accessible appointments, flexible opening hours and walk-in clinics for non-emergency issues (Q2.26, Q2.27). Discreet access to toilets and kit collection points and sample drop-off boxes in the toilet cubical (see policy category—service provision) would help overcome barriers (Q2.28, Q2.29).

#### Modelling (providing examples to aspire to/imitate)

Rather than purely giving young people information and telling them what to do (or not to do), participants suggested it could be more effective to *show* them real life examples of how to test (eg, self-sample collection; Q2.30). Providing positive models of what they should do (eg, request a test, accept a test) could also reinforce testing importance; several spoke of credible sources such as celebrities and television/movie characters (Q2.31, Q2.32). Knowing that friends also test could help shape their thinking, which could be achieved through friend referrals or encouraging friends to attend testing together (Q2.33, Q2.34).

### Policy categories

The identified themes and sub-themes for policy categories, and illustrative quotes, are provided in table 3.

#### Communication and marketing (print, electronic, telephonic or broadcast media)

Public awareness campaigns and advertisements were identified as essential for increasing awareness and normalisation, including adverts in the community (eg, universities, see table 3, Q3.1), on television and radio (Q3.2) and leaflets and posters in general practice (Q3.3). Social media was highlighted as an appropriate method for reaching young people due to frequent use (Q3.4) and could support modelling and persuasive interventions. An annual reminder letter (or phone call) sent to younger age groups (16–17 year olds) could increase awareness, help make testing a priority and normalise testing (Q3.5).

Participants wanted clear instructions for self-sampling kits, accompanied by demonstration videos via websites to overcome capability barriers (Q3.6).

#### Service provision (delivering a service)

Most would welcome testing if offered; if they have to seek it out, it is unlikely to happen—particularly for those for whom testing is not a priority (Q3.7). Providing the option of attending an alternative HCP within the practice (staff not known to patient) would encourage testing (Q3.8). Only one female participant expressed a preference for a same-sex HCP; she felt female GPs could relate to her issues better (Q3.9).

Regarding the provision of alternative sampling methods, the majority preferred urine samples over swabs (Q3.10) and self-administered swabs over HCP-administered swabs (Q3.11). Two participants preferred HCP-administered swabs to ensure samples were collected ‘*correctly’* (Q3.12). The provision of ‘*discreet’* systems in practice (test collection points, available in toilets) and self-sampling kits to complete elsewhere (at home with return via post or drop-boxes in practice) would increase testing (Q3.13, Q3.14, Q3.15, Q3.16). This would avoid face-to-face contact and potentially embarrassing situations, enabling a sense of anonymity. It was deemed essential to provide multiple options to test—one size will not fit all (Q3.17).

The introduction of online testing, via practice websites, was raised as having the potential to encourage testing (Q3.18). Not everyone wanted testing to move completely online, with some indicating they would like the physical presence of a HCP to ask questions if doing self-sampling—at home or at the practice (Q3.19).

The inclusion of chlamydia testing as part of a young person’s annual health check was raised. Similar to the NHS Health Check for over 40 year olds, a young person’s health check could assess physical health—including chlamydia and wider STI testing (Q3.20).

#### Guidelines (documents to mandate practice)

Many believed a ‘*test during all youth consultations’* policy would increase testing. Most would accept if offered during a non-sexual health consultation (Q3.21). However, three participants felt this could lead to embarrassment or judgement if chlamydia is raised ‘*out of the blue’* (Q3.22). Offers must be approached sensitively, in a non-judgmental manner (Q3.23). Several participants said standard questions could be used (Q3.24), asked of all young people and should be non-judgemental, friendly and reassuring to alleviate embarrassment and reduce stigma (Q3.25). As well as facilitating a more comfortable environment, this would increase the likelihood of them returning (Q3.26).

## Discussion

This study explored barriers to chlamydia testing in general practice for young people and identified several intervention (increasing knowledge, inducing emotional reacting, changing physical and social contexts) and implementation (communication, service delivery, guidelines for HCPs) strategies to overcome these barriers using the BCW. Barriers such as lack of awareness/knowledge, embarrassment, fear, low risk perceptions and stigma continue to be an issue for young people.^5^ The UK primary care context reduced opportunities for testing, potentially reflecting NHS financial pressures,^29^ with GP workloads surpassing ‘unsustainable levels’, due to increasing consultations and administrative tasks.^30^ ^w1^ Several underlying reasons for barriers emerged, including the concept of ‘*sexual invincibility’* as an explanation for low risk perceptions. This concept has been linked to risk-taking behaviours, including unprotected sex^w2^ and may be linked to cognitive developmental factors such as the ‘personal fable’—a young person’s belief that they are unique and, therefore, invulnerable.^w3^ Young people often believe negative health consequences will not affect them. Such cognitive immaturity declines in later adolescents; interestingly, acknowledgement of sexual invincibility was only raised by older participants (21–24 years).

Some contradictory findings emerged. Several participants felt that fear (of receiving a positive test results and long-term impact on fertility) could discourage testing. Conversely, others felt that fear tactics (imagery and statistics) could stimulate testing. The moral aspect of inducing fear for public health promotion^w4, w5^ has been questioned, as attempts to trigger negative emotions in response to infection may actually reinforce stigmatisation.

Regarding reducing barriers to increase testing, greater education and awareness were deemed important, consistent with previous literature.^5^ This could be achieved via school-based education and multiplatform public awareness campaigns, including social media leaflets, posters, TV and radio. A recent study found *Facebook* advertisements resulted in a 41% increase in chlamydia test-kit ordered online,^w6^ reflecting perhaps the high internet usage in 16–24 year olds.^w7^ However, as illustrated by previous interventional studies focusing on patient education,^w8-10^ information alone would not be enough to increase testing.^w11-13^

Testing opportunities need to be expanded via service provision, guidelines and environmental restructuring. Young people favoured universal testing offers, delivered in a non-judgemental, friendly, manner; interestingly, previous literature has found interventions which encourage HCPs to offer tests to all young people to have an impact on testing rates.^w14^ Young people also want flexible appointments with options for: (1) sample collection (direct from HCP, in toilets, online); (2) sampling method (urine, HCP-administered swab, self-administered swab with easy-to-follow instructions); (3) sample completion location (at home, in practice); (4) sample return (direct to HCP, drop-box, post). Online testing was perceived favourably, linkage to general practice websites was suggested and viewed positively, but online systems should not replace in-person contact. Similar to information provision, increasing physical opportunities may not, by itself, be enough to increase testing.

It was evident that normalisation is key to increasing testing. Normalisation would require a multipronged approach which includes increasing knowledge and providing greater opportunities and also challenging perceptions of chlamydia and STIs more generally. The cognitive developmental stage of this population should be considered; early adolescence is a time when the drive to conform is strongest,^w15^ as are feelings of invulnerability to negative health consequences.^w2, w16^ Young people need to be persuaded that testing (and treatment) is easy; it is a healthy, responsible, behaviour, which ultimately will benefit them and their partners. As well as communicating these messages via campaigns, subtle positive verbal reinforcement from HCPs and the increased visibility of others who test (credible sources such as peers and celebrities) may be effective.

### Limitations

We attempted to recruit a diverse sample, including paid adverts on three social media three, and emails to over 600 organisations. However, the majority of participants were female, White, students, educated and over 20 years old. All participants identified as cis-gender (gender identity corresponds with birth sex). The experiences of transgender/gender diverse individuals, cis-men, those with lower education, those under 20 years and other ethnicities may not be well represented. One potential explanation for the lack of diversity is that recruitment materials and strategies were developed with members of the public who were also White, cis-gender, educated, women, over 20 years of age. Future research should attempt to engage with more diverse populations from conception through to completion.^w17^

Given the sensitive nature of the subject, those who participated could differ from those who refused to take part. However, we provided the option of in-person or phone interviews to reach those who may have been reluctant to take part. Phone interviews can increase participants’ sense of anonymity regarding sensitive topics, increasing data quality.^22^ For the current study, we felt these benefits outweighed the disadvantages (lack of visual and non-verbal cues) of this method. Similar data were produced by both mediums in our study; however, a comparative methodological analysis is beyond the remit of this paper. The average length of the interviews (phone and in-person) was rather short; this could be due to participants having time to consider the aim of the research in advance (study materials were sent 1 week before interviews took place); indeed, several participants provided succinct, well thought out, responses.

Distinguishing between some BCW categories was challenging. For example, embarrassment is an emotional response, so we categorised this as automatic motivation. However, it is created by the presence of others and perceived socially (un)acceptable behaviours; hence, it could have been categorised under social opportunity. There is potential overlap between categorisations within psychological capability and reflective motivation; for example, perceived low risk (reflective motivation) could be due lack of information and awareness on the risk of transmission (psychological capability), illustrating the complex interplay between components of the first tier of the BCW. No themes were categorised under enablement, defined as ‘increasing means/reducing barriers to increase capability (beyond education and training) or opportunity (beyond environmental restructuring)’. Normalisation was not included as a distinct theme, but the means through which normalisation could be achieved fit into several other categories (eg, education, modelling, guidelines).

### Implications for research, policy and clinical practice

Any attempt to improve young people’s sexual health must recognise subpopulations. Experiences will differ due to, for example, age, employment, experiences and sexual orientation. Efforts to increase testing should focus on the youngest of young people, who were believed to be affected more by stigma, embarrassment and feelings of invulnerability and were less aware of testing importance and procedures. Several participants identified as sexual minorities; most had not disclosed their sexual orientation to their HCP due to discrimination concerns and negative judgements. Consequently, several sexual minority participants were uncomfortable with testing in general practice. This warrants further attention, particularly given the current UK situation, whereby sexual health clinics are closing due to lack of funding,^30^ almost one-fifth of opportunistic asymptomatic chlamydia testing is conducted in general practice^1^ ^w18^ and the NHS recommends that GPs ask patients aged 16 and older about their sexual orientation.^w19^

## Conclusion

At this juncture, we cannot definitively state what an effective intervention to increase chlamydia testing in general practice would consist of. This study has provided novel insights into what young people believe would be effective. Interventions need to be tailored to young people; they are a heterogeneous population. Simply providing information or opportunity will not bring about dramatic increases in testing; a structural and societal shift towards normalising chlamydia testing is required. Changing social norms will not be an easy feat.^w20, w21^ As evidenced here, a multifaceted approach is needed which challenges their young people’s perceptions of the infection (eg, that it is a serious STI), of their peers (eg, that their friends test) and of their self-identity (eg, that young people are not invincible), while simultaneously works towards providing education (eg, testing and treatment processes), greater opportunities for testing (eg, how and where tests are conducted), role models and examples of the behaviours (eg, through credible sources) and guidelines for HCPs (eg, universal testing without judgement).

This paper has provided one piece of the puzzle in developing theory-based and evidence-based interventions to increase chlamydia testing in general practice; however, further strands of evidence are required. Future research should engage primary care staff and commissioners to understand what needs to change from their perspectives and what it is realistically possible to change in the current UK primary care context. The cost-effectiveness and long-term sustainability of efforts to increase testing also require further exploration. Then, once these strands of evidence have been triangulated, the next stage will be to identity the active ingredients and mechanisms of action required to significantly influence chlamydia testing. Nonetheless, researchers, clinicians and policy makers must keep patient diversity at the forefront to ensure optimal and inclusive healthcare. Addressing the health issues affecting all young people will be a crucial part of improving national public health and eliminating STIs.

**Key messages**

- Increasing information and opportunities alone may not equate to increases in testing; a multifaceted, flexible approach is required which challenges negative perceptions of chlamydia.
- Communication and messages to encourage testing should take into account adolescent cognitive (feelings of invulnerability) and social (desires to conform) developmental factors.
- Efforts to increase testing must be inclusive and consider patient diversity; the needs of sexual and gender minorities need to be acknowledged and met.

10.1136/sextrans-2019-054309.supp4 Supplementary data
