# Supplementary material for: How to increase chlamydia testing in primary care: a qualitative exploration with young people and application of a meta-theoretical model
Source: Sex Transm Infect. 2020 May 29;96(8):571–81. doi: 10.1136/sextrans-2019-054309 (PMC7677464; doi:10.1136/sextrans-2019-054309)
Supplement: Supplementary data [file sextrans-2019-054309supp001.pdf]

GENERAL PRACTICE CHLAMYDIA TESTING FOR YOUNG PEOPLE

Supplementary File 1

Interview Topic Guide

Icebreakers

1. Can you start by telling me how you find out about this study?
   – Why did you want to take part?

2. What do you know about chlamydia?
   – Where did you learn this information? *Prompts:* school, friends, family internet.

3. Do you know where you would go to get a chlamydia test?

4. What do you think a chlamydia test involves?
   – How easy or difficult do you think a chlamydia test is to do?

5. Have you ever had a chlamydia test?
   – Can you tell me more about that experience?
   – Was it offered to you or did you seek it out? Why or why not?
   – Where/how was this offered to you?
   – How did you feel about being offered it?
   – If yes, was the result positive or negative?
   – If positive: Did you seek treatment? Did you tell any previous/current sexual partners about it? Would you get tested again in future? Why or why not?

Opportunistic testing

6. If you were offered a chlamydia test by your GP whilst in a consultation for an unrelated health issue, (for example, a sprained ankle, or the flu) how would you feel?
   – *Probe:* Can you tell me more about that?

7. If you were offered a chlamydia test by your practice nurse (at your general practice) whilst in a consultation for an unrelated health issue, how would you feel?
   – *Probe:* Can you tell me more about that?

GENERAL PRACTICE CHLAMYDIA TESTING FOR YOUNG PEOPLE

8. If you were offered a chlamydia test by your GP whilst in a consultation for a sexual or reproductive health issue, (for example, contraception) how would you feel?
 – *Probe:* Can you tell me more about that?

9. If you were offered a chlamydia test by your practice nurse (at your general practice) whilst in a for a sexual or reproductive health issue, (for example, contraception) how would you feel?
 – *Probe:* Can you tell me more about that?

Barriers

10. What do you think stops people your age from taking a chlamydia test at a GP surgery?
 – *Probe:* Can you tell me more about that?

11. What would stop you (OR has stopped you) from taking a chlamydia test?
 – *Probes:* Can you tell me more about that? Why was that? How did you feel about that?

12. You mentioned [insert barriers they bring up], how do you think we could overcome that?
 – *Probe:* Can you tell me more about that?

Facilitators

13. What do you think would make it easier for people to take a chlamydia test in general practice?
 – *Probe:* Can you tell me more about that?

14. You mentioned [insert facilitators they bring up] how do you think we could implement that?
 – *Probe:* Can you tell me more about that?

GENERAL PRACTICE CHLAMYDIA TESTING FOR YOUNG PEOPLE

15. What would make you (OR previously made you) get a chlamydia test?

- *Probes:* Can you tell me more about that? Why was that? How did you feel about that?

Closing Question

16. Is there anything else you would like to say or share?

- *Revisit:* Is there anything else we could do that would make it easier for you to take a chlamydia test at your general practice?
- Thank you very much for taking the time to give us your views. Your contribution is very important.
