# Supplementary material for: How to increase chlamydia testing in primary care: a qualitative exploration with young people and application of a meta-theoretical model
Source: Sex Transm Infect. 2020 May 29;96(8):571–81. doi: 10.1136/sextrans-2019-054309 (PMC7677464; doi:10.1136/sextrans-2019-054309)
Supplement: Supplementary data [file sextrans-2019-054309supp002.pdf]

Supplementary File 2. Thematic overview and summary of all findings

Barriers Themes and Subthemes		COM-B Components	Intervention Options				Policy Categories		
			Education	Persuasion	Environment. Restructuring	Modelling	Communicat. & Marketing	Service Provision	Guidelines
**Self-sampling**		Physical Capability					✓	✓	
**Lack of information and awareness**	Asymptomatic	Psychological Capability	✓	✓			✓		
	Risk of transmission		✓	✓			✓		
	Testing process		✓	✓			✓		
	Ease of treatment		✓	✓			✓		
	Availability in general practice		✓	✓			✓		
**Testing not a priority**		Reflective Motivation		✓			✓	✓	✓
**Perceived low risk**	Sexual invincibility		✓	✓			✓		✓
	Relationship status (monogamy)		✓	✓			✓		✓
	Belief chlamydia is not serious		✓	✓			✓		✓
**Embarrassment**	Knowing their GP	Automatic Motivation						✓	✓
	Being seen							✓	✓
	Procedure as invasive		✓				✓	✓	
	Having to take clothes off		✓				✓	✓	
**Fear**	Long term consequence			✓			✓		
	Expectations of stigma		✓			✓	✓	✓	✓
	Unknown testing processes			✓			✓		
	Positive result			✓					
**Guilt**				✓			✓		
**UK primary care context**	Strained system	Physical Opportunity			✓			✓	
	Practice registration				✓			✓	
	Getting appointments				✓			✓	
	Lacks urgency		✓		✓			✓	
	Time constraints				✓			✓	
**Location of toilet**					✓			✓	
**Stigma**	Sex taboo	Social Opportunity	✓			✓	✓	✓	✓
	Presumed promiscuity		✓			✓	✓	✓	✓
	Judgment from HCP		✓			✓	✓	✓	✓
	Judgment from receptionists		✓			✓	✓	✓	✓
	Younger age		✓			✓	✓	✓	✓
	Never tested		✓			✓	✓	✓	✓
	Sexual orientation		✓			✓	✓	✓	✓

Note: COM-B = Capability, Opportunity, Motivation Model of Behaviour
