# Supplementary material for: How to increase chlamydia testing in primary care: a qualitative exploration with young people and application of a meta-theoretical model
Source: Sex Transm Infect. 2020 May 29;96(8):571–81. doi: 10.1136/sextrans-2019-054309 (PMC7677464; doi:10.1136/sextrans-2019-054309)
Supplement: Supplementary data [file sextrans-2019-054309supp003.pdf]

GENERAL PRACTICE CHLAMYDIA TESTING FOR YOUNG PEOPLE

Supplementary File 3

Demographic characteristics of sample

**Demographic**		***n***	**%**
Ethnicity	White British	22	78.57
	White Irish	1	3.57
	Any other White background	2	7.14
	White and Black Caribbean	1	3.57
	White and Black African	1	3.57
	White and Asian	1	3.57
Sexual orientation	Heterosexual	19	67.86
	Gay or lesbian	2	7.14
	Bisexual	5	17.86
	Pansexual	2	7.14
Education level	GCSE	4	14.29
	A Level	5	17.86
	Certificate of higher education	3	10.71
	Foundation degree	2	7.14
	Undergraduate degree	12	42.86
	Postgraduate degree	2	7.14
Occupational status	Full-time employed	8	28.57
	Part-time employed	4	14.29
	Full-time student	11	39.29
	Not in education, employment or training	5	17.86
Relationship status	Not dating anyone	11	39.29
	In an open relationship	1	3.57
	Dating one person exclusively	12	42.86
	Living with romantic partner	3	10.71
	Married	1	3.57

McDonagh LK, *et al. Sex Transm Infect* 2020; 96:571–581. doi: 10.1136/sextrans-2019-054309
