# Supplementary material for: How to increase chlamydia testing in primary care: a qualitative exploration with young people and application of a meta-theoretical model
Source: Sex Transm Infect. 2020 May 29;96(8):571–81. doi: 10.1136/sextrans-2019-054309 (PMC7677464; doi:10.1136/sextrans-2019-054309)
Supplement: Supplementary data [file sextrans-2019-054309supp004.pdf]

Web Appendix 2

Additional references

w1. Hobbs FR, Bankhead C, Mukhtar T, et al. Clinical workload in UK primary care: a retrospective analysis of 100 million consultations in England, 2007–14. *The Lancet* 2016;387(10035):2323-30. doi: 10.1016/S0140-6736(16)00620-6

w2. Alberts A, Elkind D, Ginsberg S. The personal fable and risk-taking in early adolescence. *Journal of Youth and Adolescence* 2007;36(1):71-76.

w3. Elkind D. Egocentrism in adolescence. *Child Development* 1967:1025-34.

w4. Brookes G, Harvey K. Peddling a semiotics of fear: a critical examination of scare tactics and commercial strategies in public health promotion. *Journal of Social Semiotics* 2015;25(1):57-80. doi: 10.1080/10350330.2014.988920

w5. Ruiter RA, Kessels LT, Peters GJY, et al. Sixty years of fear appeal research: Current state of the evidence. *International Journal of Psychology* 2014;49(2):63-70. doi: 10.1002/ijop.12042

w6. Nadarzynski T, Burton J, Henderson K, et al. Targeted advertisement of chlamydia screening on social media: A mixed-methods analysis. *Digital Health* 2019;5:2055207619827193.

w7. Office for National Statistics. Internet users, UK: 2018. ONS: UK 2018. Available from: https://www.ons.gov.uk/businessindustryandtrade/itandinternetindustry/bulletins/internetusers/2018

w8. Bilardi JE, Sanci LA, Fairley CK, et al. The experience of providing young people attending general practice with an online risk assessment tool to assess their own sexual health risk. *BMC Infectious Diseases* 2009;9. doi: 10.1186/1471-2334-9-29

w9. Booth AR, Norman P, Goyder E, et al. Pilot study of a brief intervention based on the theory of planned behaviour and self-identity to increase chlamydia testing among

young people living in deprived areas. *British Journal of Health Psychology* 2014;19(3):636-51. doi: 10.1111/bjhp.12065

w10. Gold J, Goller J, Hellard M, et al. Impact evaluation of a youth sexually transmissible infection awareness campaign using routinely collected data sources. *Sexual Health* 2011;8(2):234-41. doi: 10.1071/sh10082

w11. Conner M, Norman P. Predicting and changing health behaviour: Research and practice with social cognition models. 3rd ed. England: Open University Press 2015.

w12. Conner M, Norman P. Health behaviour: Current issues and challenges. *Psychology & Health* 2017;32(8):895-906. doi: 10.1080/08870446.2017.1336240

w13. Ogden J. Celebrating variability and a call to limit systematisation: The example of the Behaviour Change Technique Taxonomy and the Behaviour Change Wheel. *Health Psychology Review* 2016;10(3):245-50. doi: 10.1080/17437199.2016.1190291

w14. Guy RJ, Ali H, Liu B, et al. Genital chlamydia infection in young people: A review of the evidence. Australia: The Kirby Institute, University of New South Wales 2011. Available from: Available from: https://www.acon.org.au/wp-content/uploads/2015/04/Genital-Chlamydia-Review-KIRBY-2011.pdf

w15. Knoll LJ, Magis-Weinberg L, Speekenbrink M, et al. Social influence on risk perception during adolescence. *Psychological Science* 2015;26(5):583-92. doi: 10.1177/0956797615569578

w16. Sales JM, Irwin CE. Theories of adolescent risk-taking: A biopsychosocial model. In: O'Donohue W., Benuto L., Woodward Tolle L., eds. Handbook of Adolescent Health Risk Behavior. San Francisco, CA: Springer 2009:13-29.

w17. McDonagh LK, Blomquist P, Wayal S, et al. Collaborative and consultative patient and public involvement in sexual health research: Lessons learnt from four case studies.

Sexually Transmitted Infections 2020;96(2):96-100. doi: 10.1136/sextrans-2018-053922

w18. McDonagh LK, Saunders JM, Cassell J, et al. Facilitators and barriers to chlamydia testing in general practice for young people using a theoretical model (COM-B): a systematic review protocol. *BMJ Open* 2017;7(3):e013588. doi: 10.1080/00224499.2017.1410519

w19. National Health Service England. Sexual Monitoring: Full Specification. NHS: London 2017. Available from: https://www.england.nhs.uk/publication/sexual-orientation-monitoring-full-specification/

w20. Jetten J, Haslam SA, Cruwys T, et al. Advancing the social identity approach to health and well-being: Progressing the social cure research agenda. *European Journal of Social Psychology* 2017;47(7):789-802. doi: 10.1002/ejsp.2333

w21. Smith JR, Louis WR, Tarrant M. University students' social identity and health behaviours. In: Mavor K., Platow M., Bizumic B, eds. Self and social identity in educational contexts. New York: Psychology Press 2017:159-75.
